# Curative surgery for multiple hepatocellular carcinomas after lenvatinib plus transarterial chemoembolization: a case report

**DOI:** 10.1093/jscr/rjad485

**Published:** 2023-08-26

**Authors:** Jin Shiraishi, Shinji Itoh, Takahiro Tomino, Shohei Yoshiya, Yoshihiro Nagao, Kazutoyo Morita, Hiroto Kayashima, Noboru Harada, Yasunori Ichiki, Tomoharu Yoshizumi

**Affiliations:** Department of Surgery and Science, Graduate School of Medical Sciences, Kyushu University, Fukuoka, 812-8582, Japan; Department of Surgery and Science, Graduate School of Medical Sciences, Kyushu University, Fukuoka, 812-8582, Japan; Department of Surgery and Science, Graduate School of Medical Sciences, Kyushu University, Fukuoka, 812-8582, Japan; Department of Surgery and Science, Graduate School of Medical Sciences, Kyushu University, Fukuoka, 812-8582, Japan; Department of Surgery and Science, Graduate School of Medical Sciences, Kyushu University, Fukuoka, 812-8582, Japan; Department of Surgery and Science, Graduate School of Medical Sciences, Kyushu University, Fukuoka, 812-8582, Japan; Department of Surgery and Science, Graduate School of Medical Sciences, Kyushu University, Fukuoka, 812-8582, Japan; Department of Surgery and Science, Graduate School of Medical Sciences, Kyushu University, Fukuoka, 812-8582, Japan; Department of Internal Medicine, Japan Community Health Care Organization Kyushu Hospital, Kitakyushu, 806-8501, Japan; Department of Surgery and Science, Graduate School of Medical Sciences, Kyushu University, Fukuoka, 812-8582, Japan

**Keywords:** hepatocellular carcinoma, lenvatinib, TACE

## Abstract

Surgical therapy following lenvatinib (LEN) plus transarterial chemoembolization (TACE) is a useful therapeutic option for intermediate-stage hepatocellular carcinoma (HCC). A 66-year-old man with a history of hepatitis C was detected four masses in the caudate lobe and segment 6/7 of the liver, with a maximum lesion diameter of 14 cm by computed tomography. The patient was diagnosed with intermediate-stage HCC and received LEN plus TACE. After resuming LEN for 8 weeks, computed tomography showed weakened stained areas of the tumors, and no new lesions. Thus, the patient was evaluated as having a partial response in the modified Response Evaluation Criteria in Solid Tumors. The patient underwent hepatic caudate lobectomy, partial hepatectomy of S6/7, and S6 microwave coagulation therapy for radical resection. The patient is currently alive and recurrence-free at 12 months postoperatively. In patients with multiple HCC lesions, hepatic resection combined with local therapy might be an effective treatment option.

## INTRODUCTION

Patients with intermediate-stage hepatocellular carcinoma (HCC) are diverse and the treatments are controversial. In recent years, the efficacy of molecular-targeted agents like lenvatinib (LEN) for unresectable HCC has been reported, with some studies reporting favorable results when combined with transarterial chemoembolization (TACE) [1, 2]. Furthermore, curative surgery following systemic molecular therapies is considered a useful therapeutic option for intermediate-stage HCC [3, 4].

In this report, we describe a case in which a patient received LEN plus TACE for intermediate-stage HCC and eventually underwent complete resection of a giant HCC and multiple smaller HCCs.

## CASE REPORT

**Figure 1:**
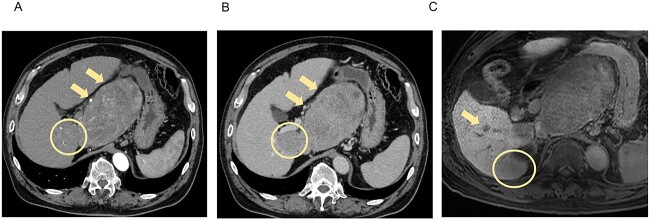
CT and MRI at the initial examination. Axial enhanced CT reveals mass lesions of 14 cm (**A**, **B**, arrow) and 3 cm (**A**, **B**, circle) in S1, with heterogenous enhancement in the arterial phase (**A**) and a washout pattern in the portal phase (**B**). Axial enhanced MRI shows the lesions in S1 and other lesions of 5 cm in S6/7 (**C**, circle) and 1 cm in S6 (**C**, arrow), which demonstrate decreased ethoxybenzyl uptake. S, segment.

A 66-year-old man presented to his previous doctor with abdominal pain. He had a medical history of hepatitis C for which he had achieved a sustained virological response with direct-acting antivirals 5 years ago. Laboratory findings showed increased concentrations of alpha-fetoprotein (AFP; 31 ng/mL) and protein induced by vitamin K absence or antagonist-II (PIVKA-II; 75 528 ng/mL), and normal liver function (Child-Pugh class A, liver damage classification A, and albumin-bilirubin index −2.87). Computed tomography (CT) and magnetic resonance imaging (MRI) at the initial examination revealed four mass lesions: a 14-cm-diameter and 3-cm-diameter lesion in segment 1 (S1), a 5-cm-diameter lesion in S6/7, and a 1-cm-diameter lesion in S6 (Fig. 1).

The patient was diagnosed with HCC (cT3N0M0, Stage IIIA, UICC, 8th edition; Barcelona Clinic Liver Cancer classification intermediate). The patient received LEN (8 mg once daily for 1 week), followed by TACE of the S6 hepatic artery (A6) and A1. One week after TACE, the patient resumed LEN (12 mg once daily) but developed fatigue; therefore, the dose of LEN was alternated between 8 and 12 mg once daily. The patient was referred to our department to evaluate the indication for hepatic resection at 4 weeks after resuming LEN, and we decided to reevaluate at 8 weeks after LEN resumption.

At 8 weeks after resuming LEN, laboratory findings showed decreased concentrations of AFP (9 ng/ml) and PIVKA-II (6636 mAU/ml), preserved liver function (Child-Pugh class A, liver damage classification A, and albumin-bilirubin index −2.62), and minimal elevation of fibrosis markers (fibrosis-4 index, 2.02; Mac-2 binding protein glycosylation isomer, 1.55 cut-off index; hyaluronic acid, 108 ng/mL; collagen type IV, 124 ng/mL; and 7S collagen, 5.4 ng/mL).

**Figure 2:**
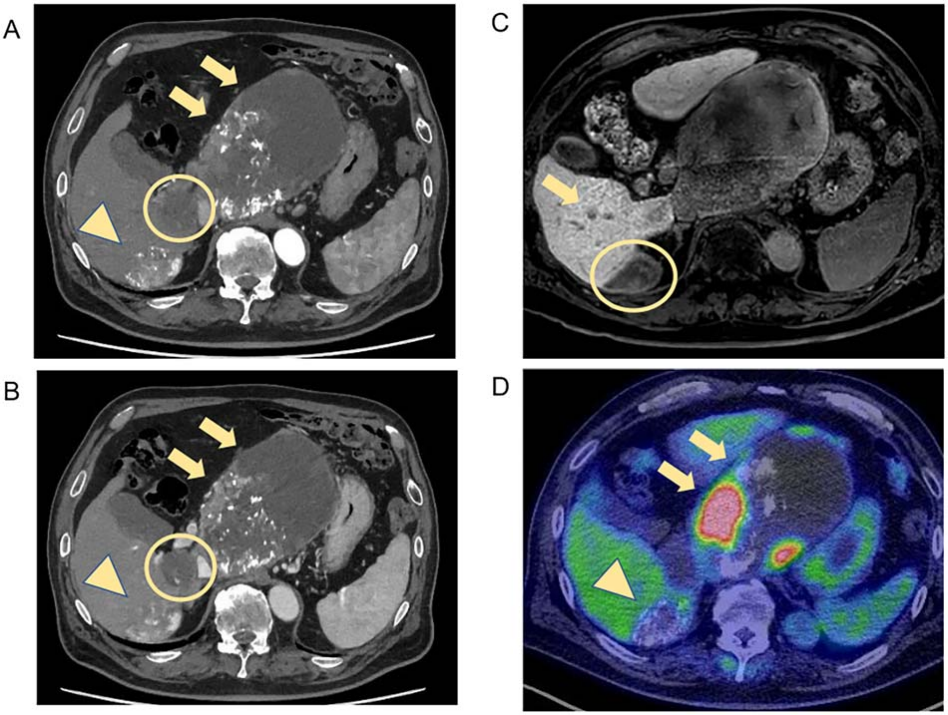
CT and MRI after LEN plus TACE. Axial enhanced CT shows weakened stained areas of the tumors in S1 (**A**, **B**, arrow, circle) and S6/7 with internal lipiodol deposition and poor enhancement (**A**, **B**, arrowhead), with weakly enhanced areas at the margins that are suspected to be viable lesions. MRI reveals lesions with extensive internal hemorrhage because of treatment in S6/7 (**C**, circle) and S6 (**C**, arrow). Positron emission tomography-CT shows the mass lesions with abnormal accumulation in liver S1 (SUV max 11.7; **D**, arrow) and S6/7 (SUV max 4.2; **D**, arrowhead). S, segment; SUV, standardized uptake value.

CT and MRI showed weakened stained areas of the S1 and S6/7 tumors with internal lipiodol deposition and poor enhancement. Positron emission tomography-CT showed no abnormal fluorodeoxyglucose accumulation suspicious for lymph node metastasis or distant metastasis (Fig. 2). The patient was evaluated as having stable disease in the Response Evaluation Criteria in Solid Tumors (RECIST) and a partial response in the modified RECIST.

He underwent hepatic caudate lobectomy, partial hepatectomy of S6/7, and microwave coagulation therapy of S6 (50 W, twice). The liver was in a state of chronic hepatitis and the S1 tumor was highly adherent to the pancreatic body; therefore, the upper margin of the pancreatic body was partially resected. A nodule, which was not detected on preoperative CT, was found in the Morrison’s fossa, and excised.

**Figure 3:**
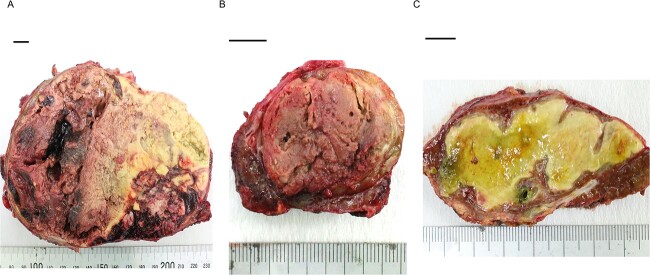
Photographs showing extensive necrosis and hemorrhage of the resected tumors after LEN and TACE. (**A**) One of the S1 tumors was a well to poorly differentiated HCC, of which 11% was viable tumor cells. (**B**) The other S1 tumor was a well to moderately differentiated HCC with 5% viable lesions. (**C**) The S6/7 tumor was a well to moderately differentiated HCC with 2% viable lesions. Scale bar = 10 mm. S, segment.

Histopathological findings showed extensive necrosis and hemorrhage of the tumors, probably because of LEN and TACE. One of the S1 tumors was a well to poorly differentiated HCC with a total size of 135 × 80 mm and viable lesion size of 39 × 31 mm (Fig. 3a). The other S1 HCC was a total size of 30 × 25 mm and a viable lesion size of 17 × 2 mm (Fig. 3b), and the S6/7 HCC was a total size of 65 × 40 mm and a viable lesion size of 15 × 2 mm (Fig. 3c). The nodule removed from the Morrison’s fossa was confirmed to be peritoneal dissemination. The noncancerous liver showed mild chronic inflammation in the portal areas with liver cirrhosis (A1, F4).

**Figure 4:**
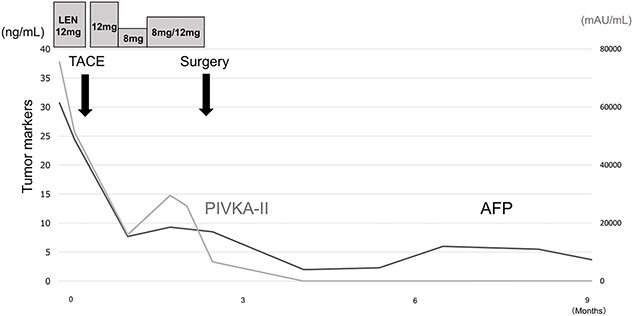
Treatment timeline with the pre- and postoperative serum AFP and PIVKA-II concentrations.

Postoperatively, tumor marker concentrations were normalized promptly after surgery (Fig. 4). The patient is currently alive at 12 months postoperatively without recurrence.

## DISCUSSION

In recent years, combined treatment with LEN plus TACE that have synergistic effects by enhancing antitumor effects has shown good results for advanced HCC [1, 5]. However, long-term LEN plus TACE has been reported to cause adverse events, and there is a risk of missed surgery opportunity because of worsening liver function [6, 7]. In the present case, curative surgery was performed after 8 weeks of LEN administration while maintaining liver function. The efficacy and optimal timing of curative surgery and systemic therapy in HCC are unclear, and further case accumulation is needed.

The effectiveness of treatment for HCC is evaluated by CT and MRI with reference to the modified RECIST [8]. In addition to imaging evaluation, follow-up of AFP and PIVKA-II concentrations is also reported to be useful for measuring treatment efficacy [9, 10]. Shindoh *et al*. [11] reported that a decrease in PIVKA-II concentration from baseline is a strong predictor of successful R0 resection after LEN treatment. In the present case, gradual decreases in AFP and PIVKA-II concentrations were observed over the course of treatment, which might help in the treatment evaluation of LEN plus TACE. Patients receiving LEN should be followed up closely by measuring tumor markers and performing imaging examinations.

A previous case report showed that surgical treatment after LEN was effective and suggested that curative surgery after LEN plus TACE might lead to prolonged survival [3]. For multinodular HCCs, including intrahepatic metastatic lesions smaller than or equal to 2 cm in diameter, hepatic resection combined with intraoperative local ablation therapy is effective [12]. In the present case, hepatic caudate lobectomy and left lobectomy were considered for the single S1 lesion; however, because of the presence of multiple lesions, hepatic caudate lobectomy was performed for the S1 giant lesion, and partial hepatic resection and intraoperative ablation therapy were performed for the S6/7 lesions. R0 resection was obtained, and the patient is alive without recurrence.

We herein reported a case of curative surgery after LEN plus TACE for intermediate-stage HCC. Hepatectomy combined with local treatment might be an effective curative therapy option for patients with multiple lesions.

## Data Availability

The data used to support the findings of this study are available from the corresponding author upon request.
